# Characterizing stroke-affected speech using F0 and duration-based features

**DOI:** 10.1038/s41598-026-40155-9

**Published:** 2026-02-15

**Authors:** M. V. S. Jyothi, Oindrila Banerjee, D. Govind, K. Samudravijaya, Akhilesh Kumar Dubey, Suryakanth V. Gangashetty, U. K. Rakesh, A. Rajeev, Jagabandhu Mishra

**Affiliations:** 1 Department of Computer Science and Engineering, Koneru Lakshmaiah Education Foundation, Vaddeswaram, Guntur, AP 522302 India; 2 Department of General Medicine, All India Institute of Medical Sciences (AIIMS), Mangalagiri, Guntur, 522302 India; 3 Department of Community and Family Medicine, All India Institute of Medical Sciences (AIIMS), Mangalagiri, Guntur, 522302 India; 4 School of Computing, University of Eastern Finland, Joensuu, North Karelia Finland

**Keywords:** Impaired speech analysis, Stroke speech analysis, F0 contour, Duration analysis, Transition and steady state regions, Acoustics, Computational neuroscience, Auditory system, Cognitive neuroscience, Cognitive control, Perception

## Abstract

This paper presents a study of pitch (F0) and duration related features of transition and non-transition regions of speech, carried out to better understand and characterize speech recorded from stroke patients. A speech corpus consisting of read speech as well as five sustained vowels, recorded from 50 stroke patients and 50 healthy speakers in a clinical environment, was developed for this purpose. A gender specific statistical analysis of F0 contour showed that median F0 of an utterance shows consistent trend in distinguishing utterances from the stroke patients and healthy control speakers across all vowel categories. The utterances from female speakers of the stroke study group tended to have a lower F0 median in comparison with that of the healthy control group. In contrast, male speakers in the stroke study group exhibited a higher F0 median compared to the control group. The durations of the transition and non-transition (steady state) regions in the utterances were estimated by an analysis of the gradient of cepstral coefficient vector as a function of frame index. The male and female speakers of the stroke study group showed shorter transition regions with higher relative transition areas as compared to those of the healthy control group. In contrast, the non-transition (steady state) regions tend to show longer duration in the stroke study group. The observed statistical trends in the F0 and duration analysis were validated using one-way ANOVA tests.

## Introduction

Speech is a natural way of communication between human beings^1–3^. Human speech production is driven by the coordination of the cognitive activities in the brain and articulators that are responsible for generating voice^4^. Therefore, impairments occurring in any of these stages affect the way in which the speech is produced, and results in dysarthria.

Dysarthria, derived from “dys” (difficulty) and “arthria” (articulation), generally refers to impaired articulation of speech sounds. Speech is produced when neuro-muscular commands are transmitted via motor cortex to various speech articulators^5–8^. Impairment due to any neurological condition significantly affects speech production. Dysarthria can also result from structural abnormalities of the speech articulators. However, the focus of this study is on characterizing speech produced by a person with dysarthria arising due to brain stroke, one of the neurological conditions.

### Background

Two primary parameters that characterize the quality of a spoken utterance, as perceived by listeners, are fundamental frequency (F0) and temporal features of the utterance^9^. F0 represents the frequency of vocal fold or glottal vibrations^9,10^. Temporal features of speech include the durations of phones, syllables, words, sentences, as well as transitions and steady-state segments. Collectively, F0 and duration-based features define the prosodic characteristics of a speech signal^11–13^. Dysarthria due to various neurological conditions is frequently associated with abnormalities in the prosody^14,15^. Consequently, there has been a growing interest in the research community to study and understand the changes in the prosody of speech produced by stroke patients vis-a-vis those of the healthy speakers.

Prosody in an utterance carries salient information collectively representing language, speaking style, speaker characteristics, emotional aspects and so on^16–21^. Therefore, most of the speech analysis studies reported in the literature were focussed on studying characteristics of prosodic features of dysarthric utterances with respect to that of the healthy control speakers^14,15,22–25^. This will enable classification of an utterance as produced by subjects having impaired voice characteristics or not. In a recent work by Narendra et al., glottal parameters were used for distinguishing Parkinson Disease (PD) patient’s voice from that of a healthy speaker^26^. Such Systems helps to provide early indications about certain neurological conditions^26^.

A perceptual study conducted by Bunton et al., has shown a significant reduction in the prosodic ratings in conversational speech of clinical groups as compared to that of neurologically normal subjects^15^. The study emphasized the degradation in the prosodic ratings even when word recognition intelligibility ratings were higher in clinical groups^15^.

Lowit-Leuschel et al. studies the variations in prosodic parameters in the dysarthric group for reading and conversational speech tasks^22^. Lowering F0, increase in the number of unstressed vowels and reduction in the unstressed vowel duration were some of the prosodic characteristics that were observed in healthy speakers conversation samples as compared to that of dysarthric group.

In a related work within the Indian context, Tanuka et al. demonstrated that using a fixed F0 segment as input to a network of one-dimensional convolutional neural networks (1D-CNN), followed by long short-term memory (LSTM) units, achieved an improved recognition performance compared to conventional mel-frequency cepstral coefficients (MFCCs) for classifying dysarthric speech^27^. Their study involved one-minute spontaneous speech samples from 38 Amyotrophic Lateral Sclerosis (ALS) patients, 45 PD patients, and healthy controls, spanning six Indian languages: Bengali, Hindi, Kannada, Odiya, Tamil, and Telugu.

Tsanas et al. proposed a classification system using a predefined set of 23 features consisting of statistical measures computed from F0, source features, jitter, shimmer and spectral features^28^.

Thoppil et al. proposed jitter, F0 breaks and frequencies of the first two formants as features to characterize dysarthria due to various neurological conditions^29^. Flatness of the F0 contour and speech rate variations were reported as the characteristics of speech utterances of patients suffering from Cerebral Palsy (CP) and ALS conditions^30^. All the aforementioned works emphasize the F0 related features in characterizing dysarthria due to various pathological conditions.

Many studies, reported in the literature, discussed the importance of using duration as a salient characteristic of dysarthric speech^31–34^. Vyshakh et al. demonstrated that duration modification of utterances in the UA speech corpus^35^ improved word recognition rates in a classical Hidden Markov Model-Gaussian Mixture Model (HMM-GMM) based isolated speech recognition system^34^. Xiong et al. explored duration modification techniques to transform dysarthric speech to normal speech (atypical speech to typical speech) for improving the speech recognition rates^31^. Banerjee et al. demonstrated that modifying duration using scale factors, determined through mean opinion score based subjective empirical studies, effectively reduced listening effort in understanding utterances recorded from a patient with Traumatic Brain Injury (TBI), a neurological condition caused by brain injury^33^. Yorkston and Buekelman reported that patients with TBI, exhibit prosodic abnormalities including slow speech rate, monotone pitch, and reduced variation in loudness. Wang et al. observed slow speaking rate, slow articulation rate, small phonation duration, larger pause duration, higher rate of change in speaking rate and lower articulation rate of change, in a comparative study carried out on the syllables and sentences recorded from 12 TBI patients and 8 healthy patients^36^. The study also reported a smaller difference in the F0 slope between stressed and unstressed words^36^. Suhas et al. reported a Diadochokinesis (DDK)-based duration analysis on monosyllabic utterances recorded from a clinical cohort consisting of 25 speakers each from ALS and PD across six languages^37^. The analysis of monosyllables (*pa*, *ta*, *ka*) and monosyllabic sequences (*pataka*, *badaga*) showed a reduced speaking rate and decreased articulatory precision in the clinical group compared to healthy controls.

### Motivation and contribution

Most of the aforesaid works reported on the prosodic analysis, were carried out in a group of dysarthric patients with various neurological conditions such as PD, ALS and CP. This paper attempts a prosodic analysis, focusing on fundamental frequency (F0) and duration, of utterances recorded from patients affected by various stroke conditions.

The goal of the analysis is to check for any specific F0 and duration characteristics observed for speech utterances recorded from stroke patients.

For the futuristic development of a clinician led stroke speech assessment system, a speech corpus was created by recording sustained vowel uttered by stroke patients and healthy speakers in the hospital clinical environments. A gender specific statistical analysis of F0 contour showed that median F0 of an utterance shows consistent trend in distinguishing utterances from the stroke patients and healthy control speakers across vowels. Similar trends were observed in the durations of phone transition and steady state regions after an analysis of prototypical bisyllabic words uttered by the two study groups.

The contributions of the paper are listed below.

- The present study focuses on stroke, a relatively less explored neurological condition.
- Proposes a joint analysis of F0 and duration parameters in stroke study and healthy control groups.

The remainder of the paper is structured as follows. “Proposed prosodic analysis approach” outlines the methods employed for F0 and duration estimation and analysis. The experimental setup, including details of the dysarthric speech corpus used for the study, is described in “Experimental setup”. “Experimental results” presents the experimental results and corresponding discussions. Finally, “Conclusions” provides a summary of the key findings and highlights directions for future work.

## Proposed prosodic analysis approach

In this section, the approach followed for prosodic analysis is described. This involves a study of statistical measures of F0 and duration measures, derived from the speech utterances of the stroke group and healthy control group.

### F0 estimation and analysis

**Fig. 1:**
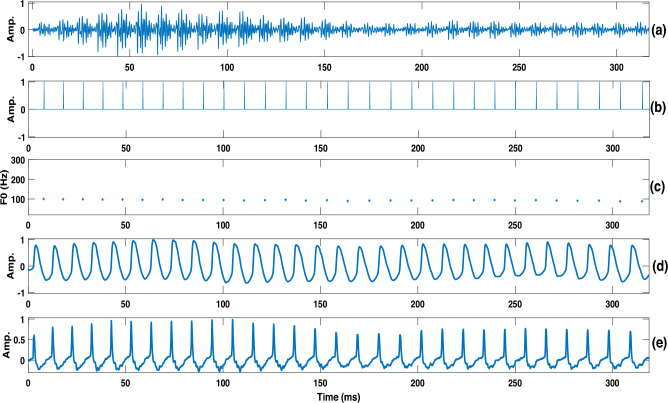
(**a**) Waveform, (**b**) epoch locations indicating the pitch marks, (**c**) instantaneous F0 derived from epoch locations, (**d**) corresponding segment of EGG and (**e**) the differenced EGG indicating ground truth pitch marks.

An epoch-based approach is employed to estimate the instantaneous fundamental frequency (F0) contour from each speech utterance^38,39^. The time instants corresponding to glottal closures are referred to as epochs^38,40,41^, and they serve as accurate markers of pitch in speech. Identifying these epochs is crucial for computing instantaneous F0, as it allows estimation of F0 for each glottal cycle. The instantaneous F0 contour is computed as the reciprocal of the time interval between successive epochs. Prior research has shown that instantaneous F0 captures finer temporal variations than conventional pitch estimation techniques, which typically provide average F0 over two to three pitch cycle^21,39^. Additionally, the epoch-based method has demonstrated greater robustness and accuracy compared to traditional techniques, as reported by Yegnanarayana et al.^38^.

Figure 1 illustrates the estimated instantaneous F0 extracted from the vowel utterance /A/ produced by a male stroke patient. Zero frequency filtering algorithm was used to estimate the Glottal Closure Instants (GCIs) and derive the instantaneous F0 contour. The recording was carried out in a clinical environment using an electroglottograph device, which simultaneously captured both speech and electro-glottogram (EGG) signals. This recording served as a test case to evaluate the reliability of the epoch-based instantaneous F0 estimation algorithm under clinical conditions. Figure 1a displays the speech signal of the vowel utterance with moderate background noise typical of hospital settings. Figure 1b shows the detected epochs or GCIs. The resulting instantaneous F0 contour is shown in Fig. 1c, derived from the epoch sequence in Fig. 1b. A comparison with the glottal cycles from the EGG waveform (Fig. 1d) reveals that the estimated F0 contour captures fine-grained temporal variations. The alignment of estimated epochs with the dominant peaks in the differenced EGG signal (Fig. 1e) demonstrates the precision and robustness of the epoch detection method, even in noisy clinical environments.

### Analysis of durations of transition and steady state regions

Many stroke patients tend to have reduced control of the muscles involved in the production of speech. This may hamper their ability to move the articulators in a systematic manner to generate normal speech. The speaking rate of stroke patients is sometimes lower than that of healthy speakers. The characteristics of the regions of speech corresponding to the change from one phone to the next phone could be slightly different from those of the healthy speakers. Duration of the transition region relative to the duration of the adjacent (relatively) steady region could be one such attribute. We carried out an analysis of the durations of transition and steady state regions of speech by the speakers of the stroke study group and the healthy control group. The speech corpus used in this study comprises speech files, each of which corresponds to a 3-word long sentence read by the speakers. The boundaries of the transition regions in speech signal were estimated following an earlier approach^42^. The transition regions in each utterance were estimated by deriving the cepstral difference curve computed from the mel frequency cepstral coefficients^43–45^. The slope of the cepstral trajectory, the cepstral difference ($\Delta C(l)$) at the $l^{th}$ frame, was calculated as shown below.

1 $$\begin{aligned} \Delta C(l)=\sum _{m=1}^{M}\sum _{k=1}^{K}k|C(m,l+k)|-C(m,l-k)| \end{aligned}$$

Here, *C*(*m*, *l*) represents the $m^{th}$ cepstral coefficient in the $l^{th}$ frame of the speech utterance, and $2K+1$ is the length of the region (number of frames) over which cepstral slope is estimated. In the present work, cepstral difference, $\Delta C(l)$ was computed by considering two frames before and two frames after the current speech frame; so, $K=2$ and $M=13$. The cepstral difference, $\Delta C(l)$, was computed for all speech frames of the utterance.

**Fig. 2:**
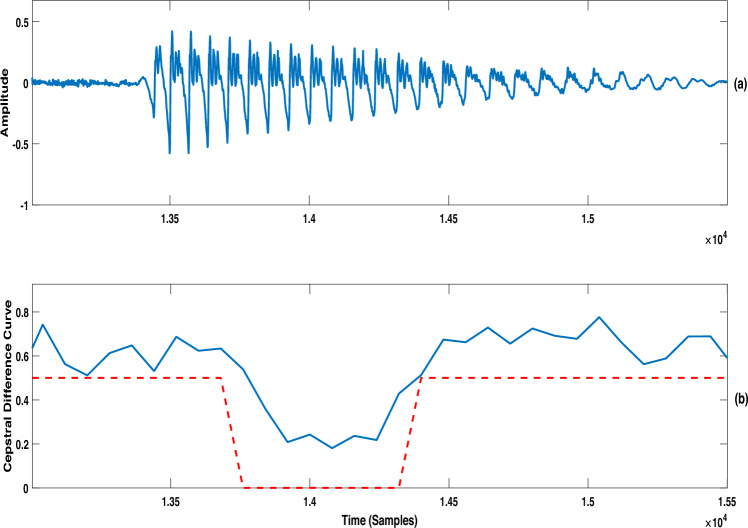
(**a**) Speech waveform of a CV syllable; (**b**) corresponding cepstral difference curve ($\Delta C(l)$ in blue color) and the identified transition regions (indicated by the red dotted line).

An interval where $\Delta C(l)$ values are higher than a threshold was identified as a transition region. The threshold was set as 50% of the maximum value of $\Delta C(l)$ in the utterance. The regions between successive transition regions were labeled as non-transition (or steady state) regions of the utterance. Figure 2 illustrates such regions of a CV syllable of an utterance selected from the phonetically balanced CMU-Arctic database^46^. The Fig. 5a shows the time waveform; Fig. 5b shows the cepstral difference curve of the utterance (in blue color), and also indicates the two transition regions (the dashed red colored curve) separated by a non-transition region.

Three features of transition regions, identified from the cepstral difference curve, were computed for further analysis. The three features are Relative Transition Duration (RTD), Relative Transition Area (RTA) and Transition Rate (TR). RTD is computed as the ratio of the total length of the estimated transition regions to the total length of the utterance. RTA is computed as the ratio of the sum of all excessive $\Delta C(l)$ values in the transition regions to the total number of frames (*L*) in the utterance. The computation of RTA is described in Eq. (2).

2 $$\begin{aligned} RTA=\frac{1}{L}\sum _{l=1}^{L}a(l) \end{aligned}$$

where

$$\begin{aligned}a(l)={\left\{ \begin{array}{ll} \Delta C(l)& \text { if } \, \, \Delta C(l)>0.5\\ 0 & \, otherwise \end{array}\right. }\end{aligned}$$

We define Transition Rate (TR) as the number of transition regions per second. TR is calculated as the ratio of the number of transition regions in the utterance to the length of the utterance in seconds. Features similar to RTD and RTA are computed for steady state regions too. These are called relative steady state duration and relative steady state area.

## Experimental setup

### The stroke speech corpus

**Fig. 3:**
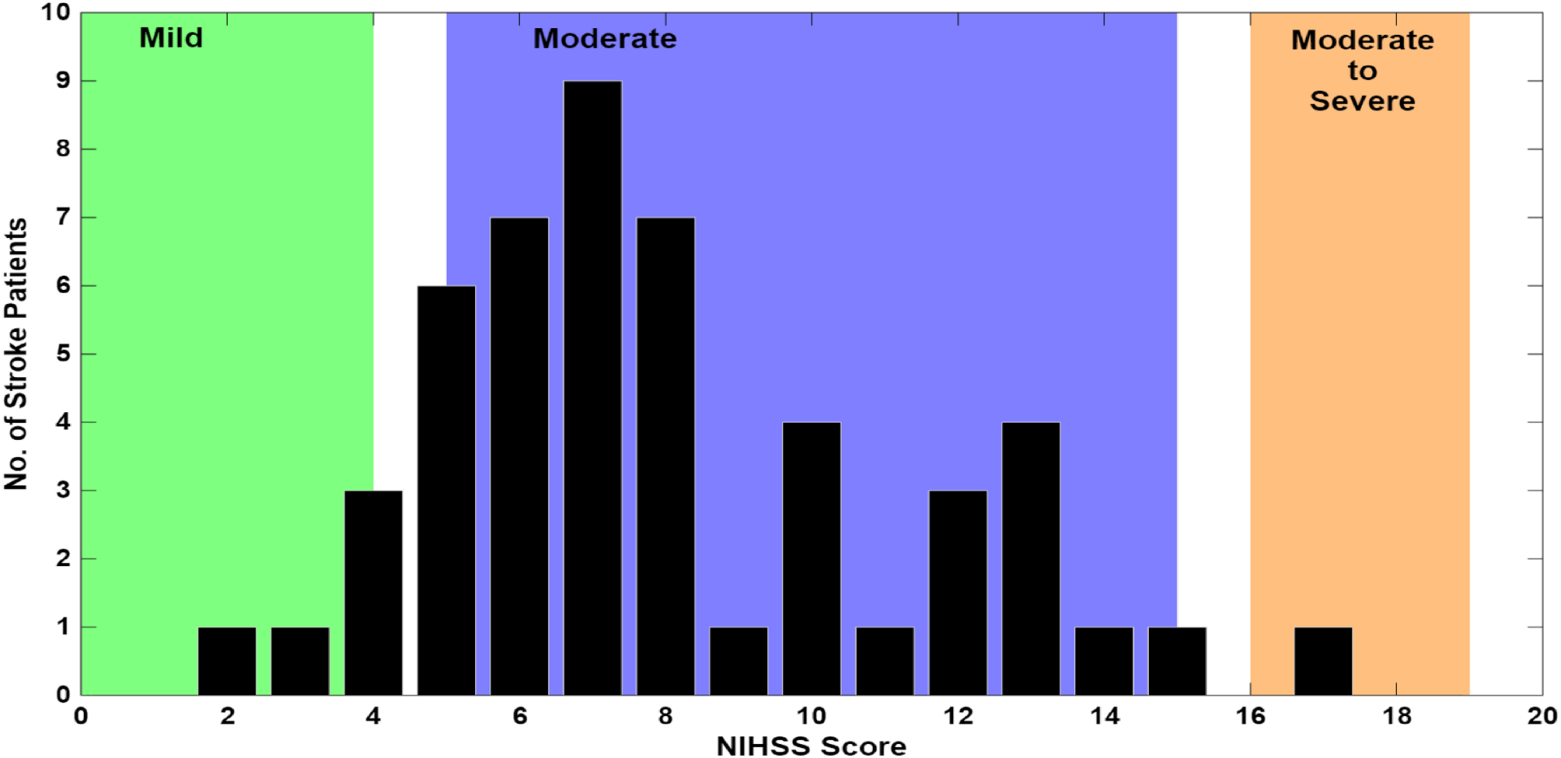
Histogram of the number of patients with various degrees of stroke in the speech corpus. Stroke severity categories are color-coded as: Mild (0–4) in green, Moderate (5–15) in blue, Moderate to Severe (16–19) in orange color. No samples are present in the Severe category with the NIHSS score in the range (20–42).

Speech was recorded in clinical environment at the stroke ward of All India Institute of Medical Sciences (AIIMS) Mangalagiri, Andhra Pradesh^47^. The data used in the present work has been collected after obtaining clearance from institute ethical committee (IEC) of AIIMS Mangalagiri with reference no: AIIMS/MG/IEC/2023-24/76. This study was conducted in accordance with the Declaration of Helsinki, and written informed consent was obtained from all participants or their immediate relatives prior to their inclusion in the study. The stroke study group consists of 30 male and 20 female stroke patients whose age ranged from 30 to 85 years, with a median age of 55. The control group of 50 healthy speakers included 36 female and 14 male speakers whose age ranged from 25 to 60 years, with the median age of 35. Both study and control group speakers are natives of Andhra Pradesh state, and speak Telugu as the first language (*L*1). The severity of stroke incident in each patient was assessed by the clinician, based on the National Institute of Health Stroke Scale (NIHSS)^48^. Among the 11 components in the NIHSS assessment, $10^{th}$ scale corresponds to the assessment related to the presence of dysarthria. The NIHSS scores range from 0 to 42. Based on the NIHSS score, stroke patients are categorized into mild (NIHHS $<5$), moderate ($5-15$), moderate to severe ($16-20$) and severe (NIHSS$>20$) categories (with no samples are present in the Severe category)^49^. Figure 3 shows the distribution of patients in the study group with respect to four stroke categories. It may be noted that about 88% of patients (44 in number), recruited in the study, were having moderate stroke severity based on the NIHSS scale.

Pilot recordings were demonstrated to the patients and their care givers in order to make them comfortable and ready for speech recording. Before the recording was started, it was ensured that the patients are not under the effect of their regular medicines as the part of their treatment. Patients were instructed to utter five vowels, /A/, /E/, /I/, /O/, /U/, in a prolonged manner. Each vowel was uttered twice. Most patients were suffering from left or right hemiparesis and hemiplegia conditions. Due to these post stroke conditions, some patients had difficulty in uttering vowels in a prolonged manner.

For an analysis of the durations of speech units in continuous speech, several sets of sentences were designed in the Telugu language. Each set contains ten phonetically rich sentences. Considering the difficulty of patients in articulating long sentences due to the impact of post-stroke conditions, each sentence is constrained to contain only three words.

Motivated by the organization of the sentences in TIMIT^50^, a popular acoustic-phonetic database, the first two sentences, S01 and S02, were kept as common across all the speakers while the remaining eight sentences were distinct for each sentence set. The last eight sentences in each set were selected to increase the overall phonetic richness of the set of ten sentences. The phonetic richness of a sentence set was measured in terms of the percentage of distinct phones of Telugu language that are present in each set of sentences. An Indic phonemizer was used to derive the sequence of phones, labeled using a Common Label Set^51^, corresponding to a Telugu sentence.

The speakers in the healthy control group read the same set of 3-word long sentences uttered by the stroke patients. To maintain uniformity in the recording environments, all speakers from the healthy control group were brought to the same hospital ward where patient recordings were performed. In both cases, speech signal was recorded using an iPad at a sampling frequency of 44.1 kHz with a resolution of 16 bits per sample. The raw audio recordings were manually segmented to generate independent utterances of each vowel category. A similar segmentation was followed for sentences. All the utterances were downsampled to 16 kHz for further analysis. Further details on the development of the clinically recorded dysarthric speech database from stroke patients can be found in the work by Banerjee et al.^52^. The speech corpus has been made publicly available for academic research via the Zenodo repository^53^. In summary, Table 1 provides an overview of the utterances used in the F0 and duration analysis, presented in this paper.

**Table 1:** Summary of the utterances used for F0 and duration analysis.

Analysis type	Utterance type	No. of speakers
		50 stroke patients
F0 analysis	Vowels (A, E, I, O, U)	50 healthy controls
		50 stroke patients
Duration analysis	Common sentences (S0 & S1)	50 healthy controls

### Details of experimental parameters

For the epoch-based F0 analysis, a window length of 10 ms was set for the estimation of GCIs from the given speech. Epochs having less than 5% of mean strength of excitations were discarded for deriving instantaneous F0 contour. The instantaneous F0 values outside the interval of [50, 300] Hz were discarded as they pertain to noisy silence regions. Sustained vowel recordings from 50 speakers of stroke study group and 50 speakers of the healthy control group were utilized for the $F_0$ analysis. In addition to all the age groups present in the dysarthric speech database, statistical analysis was compared with the matching age group of speakers within 40–50 Years for both stroke study and healthy control populations.

To identify transition and steady state segments, the cepstral difference curve was computed from the sequence of 13 MFCC coefficients extracted from a given speech utterance. MFCC coefficients were computed by setting frame size with a frame size of 20 ms and a frame shift of 10 ms and the energy coefficient was excluded. The Computation of MFCC with RASTA filtering was performed using *melfcc.m*, a utility available in audio processing toolbox of MATLAB^54^. Further, an energy threshold of 0.06 (6% of the total energy of the utterances) was set to discard the MFCC frames pertaining to the silence and short pause regions.

To reduce the influence of speaker dependency arising from differences in vocal tract length due to individual physiological variation, Vocal Tract Length Normalization (VTLN) was applied before computing the MFCC features. In VTLN, the speech spectral envelope is warped using a warping factor ($\alpha$) that is estimated separately for each speaker^55^. In this study, the warping factor for each speaker was obtained using the average *formant dispersion *($D_f$), computed with reference to a baseline $D_f$ derived from all healthy speakers of the same gender. The estimation of $D_f$, using the first three formant frequencies ($F_1$, $F_2$, and $F_3$), followed the procedure described by Fitch et al.^56^. The formant parameters $F_1$, $F_2$, and $F_3$ are determined by selecting the peaks in the linear prediction spectrum. The MATLAB implementation of the formant estimation algorithm presented in^57^ is employed in this work. The warping factor $\alpha _i$ for the $i^{th}$ speaker is then calculated as in Eq. (3).

3 $$\begin{aligned} \alpha _i=1+ \frac{1}{20}\frac{D^i_f - \mu }{\sigma } \end{aligned}$$

where $\mu$ and $\sigma$ denote the mean and standard deviation of $D_f$ values computed across all healthy speakers within the group, and these $\mu$ and $\sigma$ are used as the reference $D_f$ for the present study. The formant dispersion for any speaker, $D^i_f$, is computed according to Eq. (4).

4 $$\begin{aligned} D^i_f=\frac{1}{N}\sum _{j=1}^{N}F^j_3-F^j_1 \end{aligned}$$

where *N* represents the number of frames in the utterance. For robust formant estimation, we used sustained vowel sounds /*A*/ from our speech corpus. Once $\alpha$ was obtained for each speaker, VTLN was applied to the spectral envelope of that speaker’s utterances prior to the computation of MFCC features.

## Experimental results

### Analysis of F0 contours derived from stroke study group and healthy control group

**Fig. 4:**
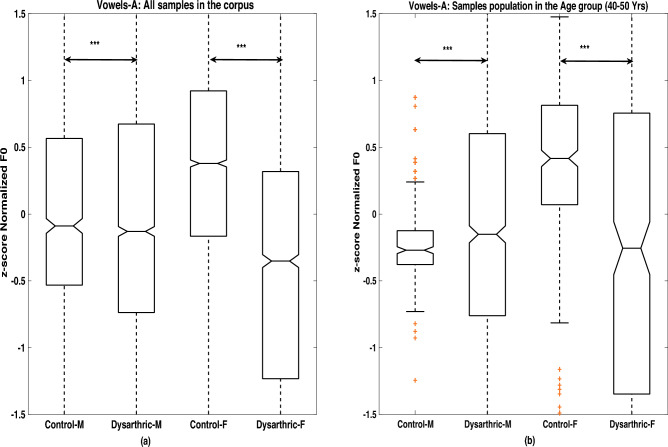
Boxplots displaying the statistical analysis of normalized F0 values derived from z-score normalization performed on the combined sample of the vowel /A/ in control and stroke groups for speakers (**a**) across all age ranges and (b) within the matched age range of 40–50 years. Significant regions are indicated using the standard notation‘***’, representing a highly significant difference between the two groups with $p < 0.001$.

For the F0 analysis, the mean, median and standard deviation measures of instantaneous F0 contours are computed. Table 2 lists these measures of F0 data of male as well as female speakers in stroke study and healthy control groups for all 5 vowels. The distributions are graphically represented as notched box plots in Fig. 4.To minimize potential speaker bias between the two groups, the pooled F0 values from both groups for each gender were z-score normalized before generating the box plots shown in Fig. 4. A gender-wise comparison of F0 values for the vowel utterance /A/ is presented across all speakers (irrespective of age) for both stroke and healthy control populations in Fig. 4a. From Fig. 4a, it can be observed that the median F0 values of female speakers in the stroke group are lower than those of female speakers in the control group. In contrast, male speakers in the stroke group exhibit higher median F0 values compared to their healthy counterparts. Table 2 lists the standard deviation of F0, computed for all vowel utterances in the stroke study and healthy control groups.

**Table 2:** Analysis of median, mean and standard deviation of $F_0$, computed from the $F_0$ contours of utterances present in the stroke study and healthy control groups.

Vowel	Gender	Stroke study group	Healthy control group	Stroke study group	Healthy control group	Stroke study group	Healthy control group
Median F0				Mean F0		Standard deviation of F0	
A	Male	133.33	133.33	141.33	140.88	40.42	37.98
	Female	168.42	202.53	165.64	200.92	50.79	46.32
E	Male	141.59	139.13	150.49	151.19	42.28	44.10
	Female	179.77	210.52	178.99	204.14	50.28	45.54
I	Male	148.14	142.85	155.24	153.23	42.28	44.22
	Female	188.23	216.21	187.19	210.38	47.61	43.87
O	Male	141.59	135.59	149.37	144.79	39.57	40.64
	Female	173.91	202.53	172.13	196.10	46.89	45.81
U	Male	150.94	150.94	157.69	161.38	42.66	46.54
	Female	186.04	213.33	184.30	208.79	49.69	45.00

In order to obtain a quantitative measure of such a trend, we define relative deviation as follows.

5 $$\begin{aligned} \zeta =\frac{{F0}_{dys}-{F0}_{ctrl}}{{F0}_{ctrl}} \end{aligned}$$

Equation 5 defines $\zeta$, the relative deviation of F0 median; here ${F0}_{dys}$ and ${F0}_{ctrl}$ represent *F*0 medians computed for stroke and control groups, respectively. A positive value of the relative deviation of F0 median indicates that the median of F0 of the stroke patients is higher than that of the healthy control group.

**Fig. 5:**
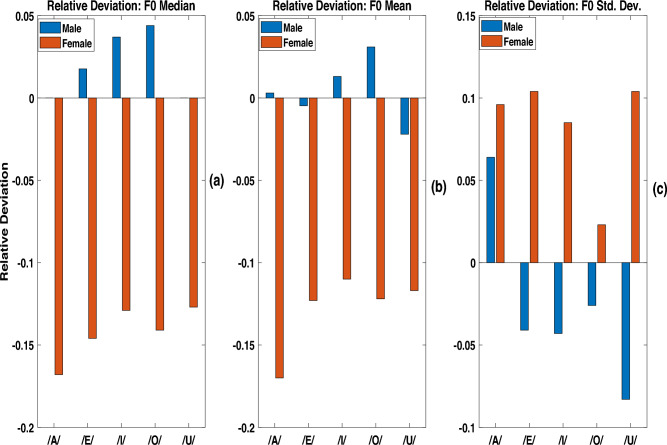
Comparison of the relative deviation, $\zeta$, of utterances in the stroke study group and the control group in male and female for (**a**) F0 median, (**b**) F0 mean and (**c**) F0 standard deviation.

The relative deviations of F0 median values of 5 vowels, computed following Eq. (5), are plotted in Fig. 5a for male speakers (blue bars) as well as female speakers (red bars). It can be seen that the relative deviations of F0 median values are positive in the case of male speakers, and negative in the case of female speakers. However, the gender dependent variation of relative deviation of F0 mean is not as distinctive as in case of F0 median, which can be seen from Fig. 5b.

In Fig. 4a, the height of the blue colored box indicates Inter Quartile Range (IQR), computed as $Q3-Q1$ where *Q*3 and *Q*1 are the third and first quartile of the dataset respectively. A visual inspection of Fig. 4a shows that the IQR of the F0 of the stroke patient group is, in general, higher than that of the healthy control group. A Similar trend is observed in case of the standard deviation of F0, as can be seen in Table 2.

Figure 5c displays the relative deviation of the standard deviation of F0 for each of the 5 vowels. The relative deviation is positive in case of female speakers, and is negative in case of male speakers for 4 vowels: /E/, /I/, /O/, and /U/. However, a reverse trend is observed in the case of vowel /A/; this observation needs further investigation.

**Fig. 6:**
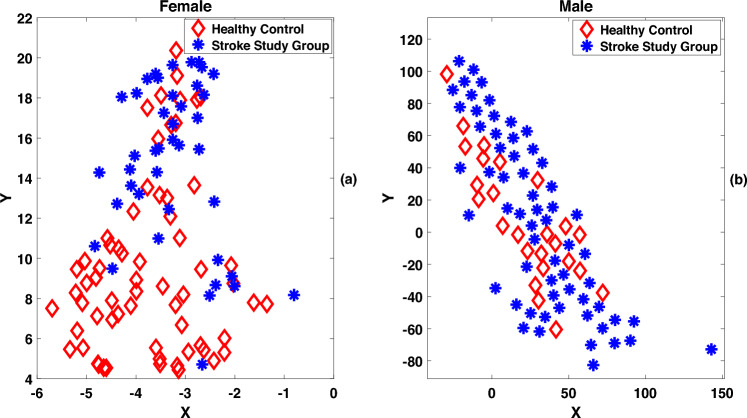
t-SNE with 2D embedding plot discriminating stroke study group and healthy control group for (**a**) male speakers and (**b**) female speakers. Here, a 3-dimensional vector comprising F0 mean, F0 median and F0 standard deviation, was projected to two dimensonal feature space (X,Y) following t-SNE approach.

The statistics of $3^{rd}$ and $4^{th}$ order such as skewness and kurtosis computed from the *F*0 contours did not show any systematic variation across all vowel categories of stroke study and control groups. Therefore, an analysis of the higher order statistics is not presented here. However, measures based on *F*0 mean, *F*0 median and *F*0 standard deviation are found to show statistical consistency for the vowel utterances present in stroke study and healthy control groups. The effectiveness of the *F*0-based three-dimensional feature vectors (mean, median, and variance of *F*0) in representing the stroke and healthy control groups is demonstrated using a t-distributed Stochastic Neighbor Embedding (2D t-SNE) plot^58,59^. In Fig. 6, although some overlap exists between the embeddings, a good separation between stroke and control groups is observable for both male and female speakers.

### F0 analysis across matched age groups of clinical study and healthy control population

Since F0 in humans changes with age, it is preferable to perform F0 analysis on age-matched groups for both clinical and control populations. The work of Eichhorn et al. showed that the middle-aged adults (40–60 years), both males and females, show only minimal variations in F0, whereas older adults (70–90 years), particularly postmenopausal women, exhibit larger variations^60^. Based on this finding, speakers in the 40–50 year age range were chosen for an F0 analysis, similar to that conducted with the entire (age independent) dataset, as described in Section 4.1. Figure 4b shows the results for the vowel /A/ under age-matched conditions. One can see that, female speakers in the stroke group exhibit lower median F0 values compared to female controls, whereas male stroke speakers show higher median F0 values than their healthy counterparts. This trend was further validated through a one-way ANOVA test conducted across all five vowels^61^. Table 3 reports the median F0 values for the control and stroke groups within the matched 40–50-year age range, along with the corresponding p-values for the five vowels. The p-values obtained from one-way ANOVA tests on z-normalized F0 values are also presented in Table 3. Statistically, male stroke patients tend to show a marginal increase in F0, while female stroke patients demonstrate a significant reduction. The exception in case of vowel /I/ needs further investigation.

**Table 3:** One-way ANOVA results for median F0 values in dysarthric and healthy control groups (aged 40–50 years), including the associated p-values and the p-values derived from z-score-normalized F0 measurements.

Vowel	Male				Female			
	Control	Dysarthric	p-value	p-value (z-norm.)	Control	Dysarthric	p-value	p-value (z-norm.)
A	114.1	117.6	0.0003	2.0E−03	188.3	160.8	0.012	3.6E−09
E	115.4	123.2	9.0E−15	1.52E−10	205.1	188.2	3.8E−5	5.2E−3
I	116.4	145.1	1.2E−20	1.19E−17	213.1	214.3	0.30	0.085
O	115.1	122.3	1.4E−20	5.40E−10	203.4	122.8	2.0E−18	1.32E−05
U	115.2	122.9	2.9E−8	1.11E−06	216.2	199.2	0.0002	1.17E−05

### Duration analysis of transition and steady state regions

**Fig. 7:**
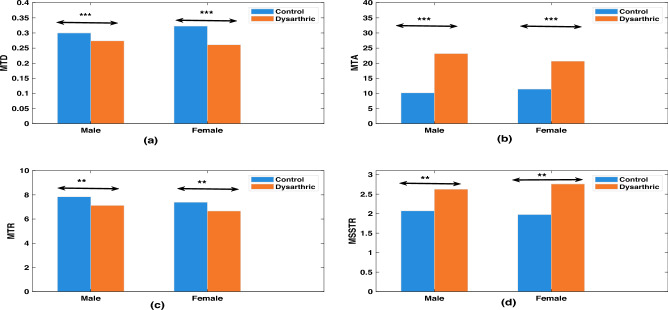
Comparison of (**a**) Mean Transition Duration (MTD), (**b**) Mean Transition Area (MTA), (**c**) Mean Transition Rate (MTR) and (**d**) ratio of Mean Steady State Duration to Mean Transition Duration (MSSTR) of the utterances at the sentence level in control and stroke study groups. The blue and orange colored bars represent data of healthy control and stroke study group respectively. The statistical significance of the differences between the two groups is denoted using standard symbols: ‘**’, ‘***’, and ‘NS’, corresponding to $p<0.05$, $p<0.001$, and no significant difference, respectively.

The results presented in the previous two subsections showed that median F0 of an utterance shows consistent trend in distinguishing utterances from the stroke patients and healthy control speakers. In this section, we present the details of similar experiments related to duration analysis. Five features, namely relative transition duration, relative transition area, transition rate, relative steady state duration and relative steady state area, computed from the utterances of the stroke study group were compared with those of the healthy control group. The details of the computation of these features are given in “Analysis of durations of transition and steady state regions”. Common sentences ‘S0’ and ‘S1’, utterred by all speakers, are considered for the duration analysis. During the recording sessions, 14 out of the 50 patients in the stroke study group were unable to speak the complete sentence in a single breath; they could speak just one word at a time. Such partial sentence recordings were excluded from the dataset used for the duration analysis experiments presented here. All the 50 speakers of healthy control group are included for the duration analysis experiments. For comparative analysis, the average of the relative transition durations of speakers in the stroke study group was computed, which is referred to as the Mean Transition Duration (MTD). The same averaging procedure was applied to three other features, which were subsequently named Mean Transition Area (MTA), Mean Transition Rate (MTR) and Mean Steady-State Duration (MSSD). In addition, the ratio of MSSD to MTD is defined as Mean Steady-State duration to Transition duration Ratio (MSSTR). As a result, we obtained the values of all five features averaged across all utterances of the stroke study group. Similar set of average values of five features were computed for the healthy control group too. This enables us to compare the values of features across the two study groups: stroke study and healthy control. Additionally, the average values of five features were calculated for gender-based subsets within each study group. This will empower us to discover any gender dependent patterns.

**Table 4:** Statistical analysis of duration measures in stroke and healthy control groups using one-way ANOVA tests.

Duration measure	Male			Female		
	Control	Dysarthric	p-value	Control	Dysarthric	p-value
MTD	0.31	0.24	1.46E−10	0.30	0.24	1.10E−07
MTA	11.73	29.54	1.60E−10	11.28	24.76	1.53E−15
MTR	7.94	6.98	0.002	7.38	6.53	0.02
MSSD	0.62	0.71	1.38E−9	0.62	0.72	5.94E−11
MSSTR	1.86	3.02	2.04E−12	2.01	3.02	5.10E−14

Figure 7 shows a gender-wise comparative analysis of the four features—MTD, MTA, MTR, and MSSTR—between the stroke group and the healthy control group. Since MFCC features extracted from all utterances of both groups have been normalized for speaker characteristics using VTLN, the duration-based measures are not affected by physiological differences across speakers. The feature MSSD, computed as the average of relative steady state duration across male and female speakers in both stroke and control groups, was not plotted since it conveys the same information as the MTD. Each subplot in the Fig. 7 contains two panels: the left panel shows results for male speakers, and the right panel shows results for female speakers. As shown in Fig. 7a, the MTD of the stroke study group is lower than that of the control group. On the other hand, MTA of the stroke study group is higher than that of the control group as can be seen from Fig. 7b. Both of these trends are gender independent. These contrasting trends are coherent, and can be reasoned as follows. The speakers of the stroke study group, in general, have weak control over the muscles responsible for the change of vocal tract configuration to cause a phone transition. As a result, changes can happen quickly or suddenly. This results in a reduction of the time needed for transition. Consequently, the transition duration decreases, on an average, as evident in Fig. 7a. Since the total change occurs during a short time, the rate of change, as reflected by the cepstral difference, $\Delta C(l)$, will be high. Such an increase in the value of $\Delta C(l)$, in case of speakers with dysarthria, will be amplified by the fact that we consider only those values of $\Delta C(l)$ which are higher than 50% of the maximum value. In case of healthy speakers, the values of $\Delta C(l)$ are likely to be marginally higher than the 50% threshold, albeit for a longer duration. Thus, the cumulative value of $\Delta C(l)$, after deduction of 0.5, within a transition region is likely to be higher in case of speakers with dysarthria in comparison with the corresponding value in case of healthy speakers. As can be seen from Fig. 7c, the MTR of the stroke study group is lower than that of the control group. Because the MTD of speakers with dysarthria is lower than that of the normal control group, the mean duration of the steady-state regions in their speech is correspondingly higher, as the steady-state and transition durations together sum to the total utterance length. A similar gender independent trend in MSSTR can be inferred from Fig. 7d.

Table 4 presents the mean values and p-values obtained from a one-way ANOVA test across all five features. It should be noted that, except for transition rate, the differences between the stroke and healthy control groups are statistically significant (*p*< 0.05).

### Discussion

From Fig. 5, one can see that all the three *F*0 contour based measures show consistent statistical trend in distinguishing the utterances of the stroke study group from those of the healthy speakers. The F0 of male dysarthric speakers shows a tendency to be higher compared to that of healthy male speakers. In contrast, the female dysarthric speakers tend to decrease their F0. The trend is more evident in the case of female speakers. One-way ANOVA test of F0 median values of dysarthric and healthy control groups (age 40–50 years) showed statistically significant differences. From the duration analysis, we observed that the speakers of the stroke study group showed a shorter mean transition duration as compared to that of the speakers in the control group. Shorter relative transition duration among utterances spoken by the stroke patients could be due to intense, reactive burst articulatory activity after prolonged reluctance, while transiting from one sound unit to other. Such behaviour of articulators in patients may be due to medical conditions such as hemiparesis (right or left) and hemiplegia observed as an after-effect of brain stroke. The longer steady state duration in the utterances of the speakers in the stroke study group appears to be correlated with the perceptual effect of slurred speech characteristics in stroke patients. The transition regions were automatically identified based on slope of MFCC feature contours. The validation of this approach by manually marked transition segments is preferable, and could be a future work.

For the duration analysis, we examined two common sentences, S0 and S1, as spoken by the stroke patients. However, depending on the severity of their condition, some patients were unable to produce complete sentences. Consequently, for the duration analysis experiments, stroke patients who did not produce sentences free of articulatory errors were excluded from the study. As a result, we were unable to obtain sufficient samples from the 40–50 age group who had spoken complete and articulatory error free sentences. Nevertheless, we performed a one-way ANOVA test on the available sentence-level utterances and reported the corresponding inferences. Sentence-level analysis, however, requires further investigation on a larger dataset that includes more dysarthric speakers producing complete, error-free sentences. For the F0 analysis, there are 12 stroke patients with dysarthria and 8 healthy controls in the selected age range of 40–50 years. Among the participants, the dysarthric group includes 10 male and 2 female speakers, while the healthy control group consists of 3 male and 5 female speakers. The relatively small sample size within this age-matched condition is a limitation of the present study. Despite the weak correlation between F0 contours and physiological characteristics, speaker-dependent variances arising from habitual and anatomical factors remain. The estimated F0 trends for both groups may thus be subject to random speaker-specific biases. A similar limitation applies to duration analysis. Since measures such as RTD, RTA, and TR are computed relative to utterance duration, they are susceptible to variations in individual speaking rates. Consequently, the current conclusions acknowledge these inherent speaker-specific limitations. Future methodological improvements should focus on explicitly modeling speaker-specific variability using approaches such as mixed-effects models. This would allow for a decoupling of speaker-related factors from linguistic or paralinguistic effects, thereby strengthening inferences about the robustness and generalizability of the F0 and duration analyses.

## Conclusions

We presented the outcomes of our study aimed at characterizing dysarthric speech in stroke-affected patients using statistical measures derived from F0 contours of sustained vowels and duration-based features of transition segments in continuous speech. To this end, we utilized a speech corpus comprising recordings of five sustained vowels from 50 stroke patients and 50 healthy control speakers, collected in clinical settings. Statistical analysis of F0 revealed that measures such as F0 mean, median, and standard deviation effectively differentiate between speech samples from stroke-affected individuals and healthy controls. A gender-specific pattern was observed in the F0 characteristics of stroke patients compared to those of healthy speakers. Notably, female stroke patients exhibited significantly lower F0 contour values, whereas male stroke patients demonstrated a marginal increase in F0 values relative to their healthy counterparts.

The duration-based feature, namely relative transition duration, relative transition area, and transition rate, showed that continuous speech of stroke patients had longer steady-state regions and shorter transition segments. This pattern contrasts with the speech of healthy control speakers. Our observations indicate that the slurred speech characteristics in stroke patients are primarily the result of the lengthening of steady-state regions in their speech.

In future, we plan to utilize features such as median F0 and mean F0, relative transition duration, relative transition area, and the duration of steady-state regions extracted from speech utterances for developing a machine learning-based system to differentiate between the speech of stroke patients and that of healthy individuals. From a feature analysis perspective, in addition to F0 and duration, future work will include the analysis of formant features representing vocal tract parameters in both stroke study and healthy control groups. Additionally, features extracted from F0 contours and segment durations hold potential for assessing the severity of stroke in affected patients. Moving forward, we aim to investigate effective strategies for integrating statistical measures of F0 and duration, alongside other relevant speech characteristics, towards the development of a clinician-guided system for stroke severity assessment.

## Data Availability

Data that support the findings of this study have been deposited in the Zenodo respository with the following reference details to access: Govind, D. Indian stroke speech corpus. Dataset on Zenodo, DOI: 10.5281/zenodo.15675906 (2025). Link to Access: https://zenodo.org/records/15675907.

## Declarations

### Approval statement

The clinical data used for the work presented in the paper has been collected after obtaining institute ethical clearance (IEC) approval from competent authorities of the medical school and hospital named All India Institute of Medical Science (AIIMS) Mangalagiri with approval reference no: AIIMS/MG/IEC/2023-24/76
